# Enhancing daily living and cognitive functions in traumatic brain injury patients through Orem’s self-care theory

**DOI:** 10.3389/fneur.2024.1449417

**Published:** 2024-08-20

**Authors:** Pei Sha, Xing Gao, Ran Yu, Ying Li, Yameng Zhang, Ziyu Zhu, Ting Wu, Chang Liu

**Affiliations:** Department of Orthopedics and Emergency Surgery, The Affiliated Hospital of Xuzhou Medical University, Xuzhou, China

**Keywords:** Orem’s self-care theory, cognitive function, neurological function, daily living ability, traumatic brain injury

## Abstract

**Introduction:**

This research seeks to investigate how early rehabilitation nursing, guided by Orem’s self-care theory, affects cognitive function, neurological function, and daily living skills in individuals who have suffered a traumatic brain injury (TBI).

**Methods:**

A study was conducted with 108 patients with traumatic brain injury who were hospitalized at our facility from January 2021 to March 2023. Based on their admission dates, the participants were separated into a control group (*n* = 56) and an observation group (*n* = 52). The control group received standard nursing care, while the observation group received a combination of conventional treatment and nursing interventions based on Orem’s self-care model. The research assessed alterations in the ability to perform daily tasks (Activities of Daily Living, ADL), neurological health (National Institutes of Health Stroke Scale, NIHSS; Glasgow Coma Scale, GCS), and cognitive abilities (Montreal Cognitive Assessment Scale, MoCA; Mini-Mental State Examination, MMSE) in both sets of participants prior to and following 4 and 8 weeks of nursing assistance.

**Results:**

Following the intervention, the group being observed showed notably increased ADL scores at 4 weeks (*p* < 0.001) and 8 weeks (*p* < 0.001) in comparison to the control group. At 4 weeks and 8 weeks after nursing, the observation group had significantly lower NIHSS scores compared to the control group (4 weeks after nursing, *p* = 0.03; 4 weeks after nursing, *p* < 0.001). GCS score comparison showed the similar results (4 weeks after nursing, *p* = 0.013; 4 weeks after nursing, *p* = 0.003). Moreover, the participants in the observation group had notably higher MoCA and MMSE scores in comparison with the control group 4 and 8 weeks after nursing (all *p* < 0.001).

**Conclusion:**

Orem’s self-care theory improves patients’ cognitive, neurological, and daily living functions of TBI patients during early rehabilitation nursing. This method helps enhance the level of care given by healthcare professionals, leading to more thorough and compassionate nursing care for patients.

## 1 Introduction

Traumatic brain injury (TBI), or head injury (HI), results from external forces impacting the head and neck, causing consciousness disturbances, memory loss, and neurological dysfunction (1). Traumatic brain injury is linked to increased levels of sickness, death, and impairment (2–4). In China, TBI prevalence is 783.3 per 100,000, with the highest mortality and disability rates (5). Within the US, there are 200 occurrences per 100,000 individuals, resulting in 500,000 fresh instances and roughly 80,000 fatalities each year (6). The mortality rates for mild, moderate, and severe traumatic brain injuries are 0.7%, 26%, and 58%, respectively, with corresponding disability rates of 10%, 66%, and 100% (5). The most common causes of injury are traffic accidents, industrial incidents, sports injuries, pregnancy complications, and military injuries (6). TBI is most common among teenagers (15–24 years old) and the elderly (15–24 years old), with males having a higher incidence and mortality rate than females (7). TBI often occurs during critical developmental periods, impairing independent living and personal growth (8). It is the most common and debilitating neurological disorder, causing motor, sensory, cognitive, and behavioral impairments, significantly reducing quality of life and imposing heavy burdens on individuals, families, and society. Despite medical advances reducing the mortality rate, many survivors face lasting disabilities, including significant cognitive impairments (9, 10).

Surgery is often used to treat traumatic brain injury (TBI), but providing quality nursing care is crucial for helping patients recover after surgery and improving their quality of life. Prominent healthcare institutions have recently integrated self-care theory into nursing methodologies to identify the optimal clinical nursing framework for TBI, aiming to improve the well-being of TBI patients, reduce the strain on patients’ families, healthcare providers, and society, and meet the ongoing pursuit of health and satisfaction (11). The self-care theory, created by the distinguished American nursing educator Dorothea Elizabeth Orem (12), was introduced to China toward the end of the 1980s. It is now acknowledged by the nursing field and has been utilized in in clinical settings, educational fields, and scientific studies. Self-care theory, self-care deficit theory, and nursing systems theory, which integrates nursing actions performed by nurses and patients’ behavior, are included in the theory. Nurses utilize three different nursing systems that are determined by the self-care requirements and capabilities of patients: the complete-compensation system, the partial-compensation system, and the educational support system. This method is widely used in providing nursing care for patients with traumatic brain injury. In complete-compensation care, nurses address all needs of elderly TBI patients, fulfilling their therapeutic self-care requirements entirely. Nursing staff and patients with traumatic brain injury collaborate to address therapeutic self-care requisites during partially compensatory nursing. The nurse assists the patients in performing self-care activities and addressing self-care deficits, adjusting their support according to patients’ needs while encouraging patients to independently manage what they can, thereby enhancing their self-care capability. TBI patients in the educational support system must acquire self-care skills and are able to engage in self-care tasks with temporary help. Nurses offer emotional assistance, practical advice, and a supportive setting. Within this framework, the nurse shifts from task execution for the patient to providing education and assistance for the patient to complete tasks autonomously. Prompt and precise instruction is essential for patients to participate in exercise therapy during early rehabilitation, following a thorough assessment of postoperative physical abilities. This involves personalized, step-by-step advancement through a combination of dynamic and static exercises, encouraging active involvement (12). Early rehabilitation exercises aim to improve muscle strength, boost joint flexibility, and recover function in the lumbar region and lower limbs. The initial nursing model for rehabilitation, which is founded on Orem’s self-care theory, seeks to facilitate the rehabilitation of motor function and activities of daily living in patients with traumatic brain injury through the early implementation of rehabilitation nursing within this theoretical framework (13). This study investigates the impact of Orem’s self-care theory combined with early rehabilitation nursing, on cognitive function, neurological function, and daily living abilities in TBI patients. This research examines how early rehabilitation nursing, based on Orem’s self-care theory, affects the cognitive function, neurological function, and daily living ability of individuals with traumatic brain injuries.

## 2 Patients and methods

### 2.1 Patients

**Figure 1:**
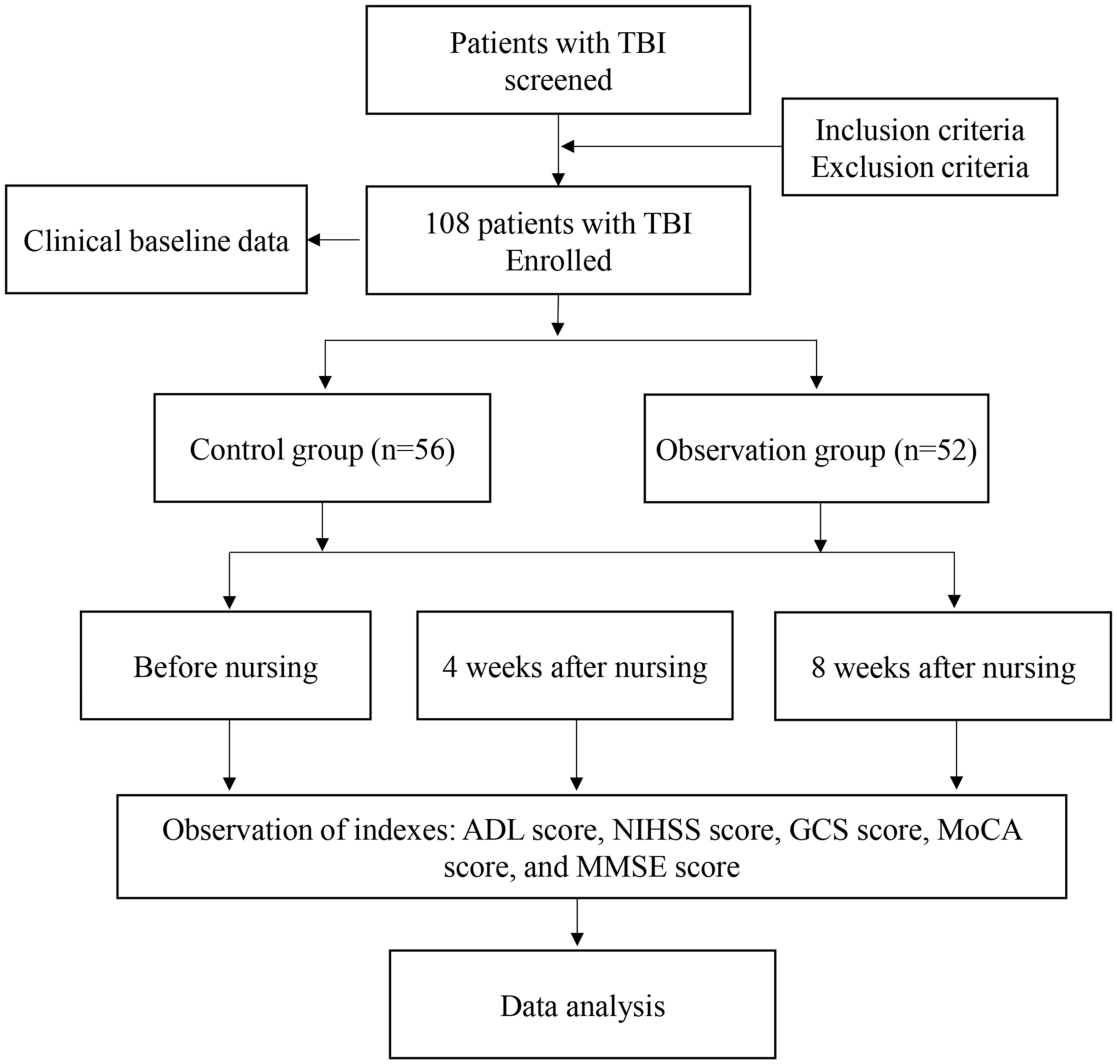
Flowchart of the study.

This study included 108 TBI patients admitted to our hospital from January 2021 to March 2023. Based on the time of admission, the participants were divided into a control group (*n* = 56) and an observation group (*n* = 52) based on admission time. After obtaining approval from the hospital’s ethics committee, the research was carried out. Criteria for inclusion in the study were as follows: (a) definite diagnosis of TBI confirmed by computed tomography scan (CT) or magnetic resonance imaging scan (MRI) according to the guidelines; (b) age ranging from 14 to 60 years; (c) Glasgow Coma Scale (GCS) score above 8; (d) Montreal Cognitive Assessment (MoCA) score below 26; (e) absence of previous cognitive impairment or mental health disorders. The exclusion criteria for the analysis included: (a) individuals who were either younger than 14 or older than 60; (b) had unstable vital signs; (c) MoCA score equal to or above 26; (d) had significant language, vision, or hearing issues, or bilateral upper limb dysfunction that hindered testing; (e) had a history of mental illness or suspected cognitive impairment prior to TBI; (f) had a history of Acute myocardial infarction, advanced hepatic or renal impairment, severe infectious pathology, or uncontrolled diabetes mellitus; (g) unconsciousness. The flowchart of this study was shown in Figure 1.

### 2.2 Treatment methods

Firstly, all nursing staff received relevant training before the start of the study and doctor-patient conflicts were avoided during the research process. The control group received standard nursing care and rehabilitation, which included anti-muscle contracture exercises, muscle strength training for flexion and extension, walking practice, and joint impedance exercises. The Orem Self-care model was applied to the observation group based on traditional nursing methods used for TBI patients in the control group, taking into account their Activities of Daily Living (ADL) scores. Patients who had ADL scores less than or equal to 40 were given a complete-compensation system, while those who scored between 40 and 60 were treated with a partial-compensation system. Patients who scored above 60 were offered an educational support system.

Patient ADL evaluation is categorized as follows: (1) Complete-compensation is assigned to patients who rely entirely on others for daily activities. Nursing staff direct patients in proper limb positioning and passive exercises to ensure correct posture. This includes muscle massages and passive joint movements to prevent atrophy and spasms. Assisted activities in bed, such as arm-raising, bridge exercises, turning, and sitting training, are facilitated to build muscle strength, laying the groundwork for ADL recovery. Attention is given to patient nutrition, regular reminders for urination and defecation, and comprehensive skin and wellness maintenance. (2) Partial-compensation is aimed at individuals who possess some level of independent living skills. Patients are encouraged to build confidence and gradually regain daily activities for the affected limb. Dressing guidance includes starting with the affected limb first. For eating, washing, grooming, and shaving, patients are initially trained in one-handed techniques, progressing to two-handed operations. Detailed explanations and demonstrations help patients master the skills, with staff providing affirmations and praise for progress. Guidance on assistive devices, such as tap-twisting towels and using bath stools, is provided to improve self-care abilities through repeated practice. (3) Patients who are able to handle most of their daily needs will benefit from the educational support system. Nurses provide both theoretical and hands-on training, motivating patients to engage in household tasks that align with their abilities in order to enhance their feelings of accomplishment and self-esteem. Training includes tasks like cleaning, making beds, sweeping, washing and drying clothes, and food preparation activities such as washing, chopping vegetables, and dishwashing.

### 2.3 Observation of indexes

(1) We evaluated the daily activities of all participants by utilizing the Barthel Index Scale to measure their ability to perform Activities of Daily Living (ADL). Evaluations were conducted before nursing care, and then again after nursing care at 4 and 8 weeks. The Barthel Index (BI) scores spanned from 0 to 100, with lower values denoting greater dysfunction in patients (14). (2) Changes in neurological function were assessed by comparing patients’ scores on the National Institutes of Health Stroke Scale (NIHSS) and Glasgow Coma Index (GCS) before and after nursing care at 4 and 8 weeks. The NIHSS scores varied between 0 and 42, where lower scores denoted less nerve damage (15). With a total GCS score of 15, a higher score signified a milder state of unconsciousness. (3) Changes in cognitive function were assessed in patients using the Montreal Cognitive Assessment Scale (MoCA) (16, 17) in conjunction with the Mini-Mental State Examination (MMSE) before and after undergoing nursing care for 4 and 8 weeks. The MoCA has a maximum score of 30, indicating higher scores are associated with improved cognitive function (18). Likewise, the MMSE scores vary between 0 and 30, with higher scores reflecting superior cognitive functions (19).

### 2.4 Statistical analysis

Data analysis and processing were carried out utilizing SPSS 24.0 statistical software. The quantitative data were presented as mean ± standard deviation (SD). Comparisons of quantitative data were executed using the *t*-test, while comparisons of categorical data were assessed via the χ^2^ test. A significance level of *p* < 0.05 was considered statistically significant.

## 3 Results

### 3.1 Clinical data

The clinical data did not show a statistically significant difference between the two patient groups (*p* > 0.05; Table 1).

**Table 1:** Comparison of clinical data between two groups of patients.

Group	*N*	Gender		Age(years)	Education (years)		Course of disease(days)	GCS scores	Treatment	
		Male	Female		≤12	>12			Surgery	Non-surgery
Control group	56	33	23	36.67 ± 6.23	39	17	28.45 ± 4.50	10.96 ± 1.55	24	32
Observation group	52	23	29	35.88 ± 5.21	32	20	29.06 ± 4.67	10.67 ± 1.47	28	24
*t/*χ^2^		2.33		1.48	0.79		0.69	1.01	1.30	
*P*		0.13		0.18	0.38		0.49	0.32	0.25	

### 3.2 The alterations in daily living abilities during treatment

Initially, we assessed the ADL ratings of both groups. Prior to the commencement of nursing care, no statistically significant difference was observed (*p* = 0.18). Following the nursing intervention, ADL scores in both groups demonstrated an improvement. The ADL scores of the observation group were significantly higher than those of the control group at both 4 weeks (*p* < 0.001) and 8 weeks (*p* < 0.001) after receiving nursing care (Table 2).

**Table 2:** Comparison of ADL scores before and after nursing in the two groups.

Group	*N*	Before nursing	4 weeks after nursing	8 weeks after nursing
Control group	56	42.16 ± 6.99	50.43 ± 6.43	58.02 ± 5.39
Observation group	52	43.94 ± 6.71	55.33 ± 7.04	65.92 ± 6.79
*t*		1.35	3.78	6.72
*P*		0.18	<0.001	<0.001

### 3.3 The alterations in neurological function during treatment

Next, we evaluated the NIHSS ratings of both groups. Before nursing, there exhibited no significant difference (*p* = 0.52). After nursing, the NIHSS scores of both groups decreased. The observation group showed a significantly lower NIHSS score compared to the control group at 4 weeks (*p* = 0.03) and 8 weeks (*p* < 0.001) post-nursing, as shown in Table 3. GCS score comparison showed the similar results (before nursing, *p* = 0.32; 4 weeks after nursing, *p* = 0.013; 4 weeks after nursing, *p* = 0.003; Table 4).

**Table 3:** Comparison of NIHSS scores before and after care in the two groups.

Group	*N*	Before nursing	4 weeks after nursing	8 weeks after nursing
Control group	56	29.88 ± 5.58	27.09 ± 5.33	23.88 ± 5.20
Observation group	52	30.58 ± 5.68	24.87 ± 5.18	19.78 ± 4.99
*t*		0.65	2.20	4.20
*P*		0.52	0.03	<0.001

**Table 4:** Comparison of GCS scores before and after care in the two groups.

Group	*N*	Before nursing	4 weeks after nursing	8 weeks after nursing
Control group	56	10.96 ± 1.55	11.95 ± 1.51	12.88 ± 1.38
Observation group	52	10.67 ± 1.47	12.63 ± 1.30	13.62 ± 1.16
*t*		1.01	2.53	3.01
*P*		0.32	0.013	0.003

### 3.4 The alterations in cognitive function during treatment

Next, we evaluated the MoCA scores of both groups. Prior to nursing care, there was no significant difference (*p* = 0.22) between the groups. Following the nursing interventions, both groups showed an increase in their MoCA scores. The observation group had significantly higher MoCA scores compared to the control group at both 4 weeks (*p* < 0.001) and 8 weeks (*p* < 0.001) post nursing care (Table 5). Then, we compared the MMSE scores. Prior to the nursing care, there was no significant difference (*p* = 0.28) between the groups. After the care was provided, both groups experienced an improvement in their MMSE scores. The observation group showed significantly higher MMSE scores in contrast to the control group at both 4 weeks (*p* < 0.001) and 8 weeks (*p* < 0.001) post nursing care (Table 6).

**Table 5:** Comparison of MoCA scores before and after nursing in two groups.

Group	*N*	Before nursing	4 weeks after nursing	8 weeks after nursing
Control group	56	19.54 ± 2.94	21.59 ± 2.67	23.20 ± 2.16
Observation group	52	18.79 ± 3.33	23.48 ± 2.51	25.54 ± 1.84
*t*		1.24	3.79	6.04
*P*		0.22	<0.001	<0.001

**Table 6:** Comparison of MMSE scores before and after nursing in two groups.

Group	*N*	Before nursing	4 weeks after nursing	8 weeks after nursing
Control group	56	17.95 ± 3.08	19.73 ± 2.93	21.11 ± 2.78
Observation group	52	17.33 ± 2.81	22.13 ± 2.35	24.96 ± 2.21
*t*		1.09	4.68	7.94
*P*		0.28	<0.001	<0.001

## 4 Discussion

TBI results from external force and can cause consciousness disturbances, memory issues, and neurological impairments (20–22). Studies have indicated that traumatic brain injury is distinguished by elevated levels of occurrence, death, and impairment (23). It is the most prevalent and debilitating condition among neurological disorders (24, 25). Individuals with traumatic brain injury (TBI) may encounter different levels of motor and sensory issues, as well as challenges in thinking, understanding, communication, daily tasks, and social connections, resulting in substantial disability and a profound effect on their overall well-being (26, 27). This condition imposes a substantial burden on individuals, families, and society. Due to the quick progress in transportation, the frequency of TBI cases is increasing (28). Despite significant medical progress in TBI treatment, reducing the overall mortality rate from 50% 30 years ago to approximately 30% today (29, 30), the emphasis on life-saving often overshadows the importance of functional rehabilitation, resulting in high disability rates among survivors. These disabilities often include cognitive, language, and physical impairments (31). A meta-analysis report endorsed the efficacy of early rehabilitation techniques for patients with stroke and traumatic brain injuries (32). The evidence suggests that perceptual rehabilitation training post-stroke and attention, memory, and executive function rehabilitation post-TBI lead to functional improvements (33). American researchers conducted a review of literature from 1998 to 2002 on the effectiveness of limb rehabilitation in patients with acquired brain injury, offering specific practical recommendations and guidelines (34). Previous meta-analyses have indicated that the impact of attention rehabilitation post-TBI and the efficacy of visuospatial therapy in addressing unilateral neglect subsequent to stroke is supported by ample evidence, with factors such as cognitive impairment type, disease progression, TBI subtype, and age playing a role (35, 36). The increasing focus on limb functional rehabilitation in China, following trends in Europe, the United States, and other advanced nations, is leading to a growing recognition of its positive effects on recovery, establishing limb rehabilitation as a key priority (35).

After a traumatic brain injury, the physical harm caused to brain tissue by outside forces, combined with an ensuing inflammatory reaction, often results in widespread neural fiber damage, resulting in substantial impairment of brain tissue (37–39). Therefore, when it comes to cerebrovascular conditions, the brain shows flexibility, as the cerebral cortex can adjust its structure and function in response to ongoing external influences. Intensive learning and training post-TBI can facilitate new connections between the dendrites and axons of neurons, or enable adjacent undamaged brain regions to take over the functions of the injured areas, thereby promoting behavioral changes (37). Therefore, early-stage rehabilitation training post-TBI can activate latent pathways and dormant synapses, prompting axonal sprouting and synapse formation, ultimately leading to synaptic regeneration and functional brain repair, reducing neurological disability. Current clinical cognitive rehabilitation encompasses various approaches, including occupational therapy, computer-based cognitive exercises, virtual reality techniques, and tele-rehabilitation for cognitive improvement (40). Given that TBI patients have a diminished capacity to process information compared to healthy individuals, integrating effective psychological and behavioral strategies into rehabilitation is crucial to enhance their information processing and absorption abilities. This makes early rehabilitation care, administered by nurses, essential for TBI patients. Involving both family members and therapists, cognitive rehabilitation helps patients integrate their mental and behavioral patterns, allowing them to analyze and synthesize information through repetitive training, ultimately enhancing their quality of life Cognitive rehabilitation involving the participation of family members and therapists integrates the patients’ mental and behavioral patterns, enabling them to synthesize and analyze information through repeated training, thereby improving their quality of life (41).

Dorothea Orem, a nursing theorist from America, first introduced Orem’s self-care theory (42). Self-care refers to an individual’s actions to maintain life, health, growth, development, and well-being by meeting their self-care needs. In the past few years, there have been significant developments in the study and implementation of self-care practices, especially in relation to long-term conditions like COPD, asthma, heart failure, diabetes, high blood pressure, and paralysis (42). Studies indicate that self-nursing interventions enable patients to acquire relevant TBI knowledge, understand their self-worth correctly, adapt to disease-induced changes, and actively engage in their rehabilitation, ultimately enhancing their quality of life. Orem’s theory of self-care highlights the capacity of individuals to take care of themselves, in line with the holistic nursing principle of focusing on the patient’s needs. Focusing on enhancing the person’s overall health and physical resilience, this method is crucial in managing illnesses (43). TBI patients received care through the application of Orem’s self-care theory in early rehabilitation nursing. By dynamically assessing patients’ self-care abilities according to their specific conditions and disease stages, various nursing systems were implemented. The self-care models were developed for individuals at different stages, ensuring that nursing care was appropriately valued and compensated based on patients’ self-care capabilities. This method assists individuals in comprehending their condition, gaining pertinent self-care information, reducing complications, enhancing limb functionality and survival abilities, increasing self-assurance, and adjusting well to both internal and external environmental changes, ultimately improving social adaptability and overall quality of life. Throughout their hospital stay, patients with traumatic brain injuries are actively engaged in making health decisions and participating in nursing care, with the goal of promoting involvement from the patients as well as their family members during the process of nursing. This involvement activates the excitement of patients, uncovers their ability to take care of themselves, and enhances their personal drive, showcasing their inherent worth and capability for self-management. Additionally, it fosters stronger nurse–patient relationships, increases job satisfaction, and enhances communication and mutual understanding, promoting a harmonious nurse–patient dynamic (44). However, this study has some limitations. Firstly, a small, single-center sample size would introduce potential bias. Secondly, this study only observed the effects after 4 and 8 weeks of nursing, lacking evaluation of long-term effects. Upcoming studies will require collaboration among multiple centers and large sample sizes to obtain more meaningful results.

In conclusion, implementing Orem’s self-care theory in early rehabilitation nursing for individuals with traumatic brain injury can lead to notable enhancements in cognitive function, neurological function, and daily living skills, making it a highly recommended approach for clinical practice.

## Data Availability

The raw data supporting the conclusions of this article will be made available by the authors, without undue reservation.
